# Chronic Right Ventricular Pacing in the Heart Failure Population

**DOI:** 10.1007/s11897-018-0376-x

**Published:** 2018-02-12

**Authors:** Justin Gould, Benjamin Sieniewicz, Bradley Porter, Baldeep Sidhu, Christopher A. Rinaldi

**Affiliations:** 1 King’s College London, London, UK; 2 Guy’s and St Thomas’ NHS Foundation Trust, London, UK

**Keywords:** Chronic right ventricular pacing, Cardiac resynchronization therapy, CRT, Biventricular, Heart failure

## Abstract

**Purpose of Review:**

We review the trials that have demonstrated potentially harmful effects from right ventricular (RV) apical pacing as well as reviewing the evidence of alternative RV pacing sites and cardiac resynchronization therapy (CRT) for patients who have heart failure and atrioventricular (AV) block.

**Recent Findings:**

The role of CRT in patients with AV block and impaired left ventricular function remains an important consideration. The BLOCK HF trial demonstrated better outcomes with CRT pacing over RV pacing in patients with left ventricular systolic dysfunction (LVSD) and AV block who were expected to have a high RV pacing burden, but failed to demonstrate a mortality benefit.

**Summary:**

CRT seems to have a beneficial effect on left ventricular reverse remodeling, systolic function, and clinical outcomes in patients with New York Heart Association (NYHA) functional class I–III heart failure, moderate to severe LVSD, and AV block compared to RV pacing. However, it is less clear whether there is a similar benefit from CRT in patients with a high percentage of RV pacing who have normal or mild LVSD in the treatment of AV block.

## Introduction

Right ventricular (RV) pacing is an important and effective treatment in patients with atrioventricular (AV) block. RV pacing restores the heart rate to a pre-determined rate; however, a high RV apical pacing percentage/burden may promote left ventricular systolic dysfunction (LVSD) [1–9]. Alternative RV pacing sites have been explored to combat this problem as well as investigating cardiac resynchronization therapy (CRT) in patients with AV block with mild to severe heart failure. CRT is an effective therapy to improve symptoms and reduce mortality in patients with dyssynchronous heart failure [10]. CRT has consistently demonstrated benefit in treating patients with systolic heart failure and interventricular conduction delay, typically with left bundle branch block (LBBB) [11–13]. However, numerous trials have used moderate and high-degree AV block in their exclusion criteria to independently evaluate the effects of CRT without the potential cofounding detrimental effects of RV pacing [14••]. Notwithstanding, several studies have demonstrated the deleterious effects of RV apical pacing, and therefore alternative RV pacing sites have been explored as well as using CRT for patients with narrow QRS and/or mild to moderate heart failure in patients who are predicted to require a significant amount of RV pacing [15]. In the present review, we review the trials that have demonstrated potentially harmful effects from RV apical pacing as well as reviewing the evidence of alternative RV pacing sites and CRT for patients who have heart failure and AV bock (Block HF and BioPace trials).

## Chronic Right Ventricular Pacing and Its Deleterious Effects

Single- or dual-chamber RV pacing is the mainstay of treatment for symptomatic AV block. However, there is increasing evidence of potential adverse effects with chronic RV apical pacing secondary to mechanical and electrical dyssynchrony [16, 17]. The detrimental effects from chronic RV pacing including the manifestation of heart failure, adverse left ventricular (LV) remodeling, and LVSD have repeatedly been reported [1–9]. These include a wide array of structural changes incorporating left atrial and LV remodeling, LV wall thickness, and functional mitral regurgitation [18–21]. In patients with complete AV block, both cellular and intracellular changes have been described including degenerative fibrosis [22]. The Dual Chamber and Implantable Defibrillator (DAVID) trial enrolled patients undergoing implantable cardioverter-defibrillator (ICD) implantation without bradycardia or AV block and randomized them to either DDD pacing at 70 beats/min or VVI backup pacing at 40 beats/min. The DAVID trial identified significantly more heart failure and cardiovascular events in the DDD group with a higher percentage of RV apical pacing [5]. Similarly, the Mode Selection Trial (MOST) demonstrated that RV apical pacing may lead to heart failure; however, the loss of AV synchrony itself was shown to probably be less important. The MOST investigators found a significantly increased risk of heart failure events in both single- and dual-chamber pacing modes, with a threshold for adverse outcomes with an RV pacing percentage greater than 40% [15, 23]. The Multicenter Automated Defibrillator Implantation Trial (MADIT II) randomized patients with ischemic cardiomyopathy and left ventricular ejection fraction (LVEF) ≤ 30% to ICD therapy versus (vs.) conventional medical therapy. MADIT II showed that ICD therapy reduced total mortality [24]. A subsequent subanalysis showed that patients with a high RV pacing percentage had a significantly increased risk of new or worsening heart failure [24, 25•]. The potentially harmful effects of long-term RV pacing may occur in patients with both preserved and reduced LV systolic function; however, they are more prominent in patients with a reduced LVEF at baseline. The true incidence of LV remodeling secondary to RV apical pacing is not known; however, it is widely recognized to occur where RV pacing is > 40% of the time [23]. However, there are some pacing-dependent patients who have 100% RV pacing who do not develop LV dysfunction for reasons that are unknown [26].

## Pathophysiology of the Detrimental Effects of Right Ventricular Pacing

Several clinical studies have established the potential adverse effects of chronic RV pacing on LV function. The exact pathophysiological process underpinning the deleterious effects from chronic RV pacing is not clear. RV apical pacing may have adverse effects on hemodynamics, remodeling, mechanical function, myocardial metabolism, and perfusion due to mechanical and electrical dyssynchrony [15, 27, 28]. An LBBB-type pattern is widely recognized to develop immediately following RV apical pacing. Early activation of the RV apex subsequently causes mechanical dyssynchrony as well as increasing early systolic shortening which results in pre-stretch of the late-activated regions and subsequent premature relaxation [15, 29, 30]. As a result, changes in LV mechanical and electrical activation due to RV apical pacing may lead to a decrease in cardiac output as well as intraventricular and interventricular dyssynchrony resulting in LVSD. This has been demonstrated in a number of studies using Doppler and strain analysis on 2-dimensional (2D) and 3-dimensional (3D) transthoracic echocardiogram (TTE) [8, 15, 27, 28, 30–34]. In addition, reduced ventricular diastole and increased ventricular systole may lead to reduced coronary perfusion [15]. Interestingly, with chronic RV apical pacing, up to 65% of patients have been found to have myocardial perfusion defects in the pacing region in the absence of flow-limiting coronary artery disease [35–37].

## Alternate Right Ventricular Pacing Sites

The advent and safety of active fixation leads has facilitated the exploration of alternatives to the traditional apical RV pacing site. However, using other RV pacing sites such as RV outflow tract and septal pacing on their own may not be sufficient to circumvent the detrimental effects of chronic RV pacing. This might be explained by technical difficulties with lead placement as well as no clear evidence of superiority of RV high septal pacing, not to mention evidence of worsening LVEF with any RV pacing site [15]. The PROTECT-PACE study randomized 240 patients with high-grade AV block requiring > 90% ventricular pacing and preserved baseline LVEF > 50%, to receive pacing at the RV apex (*n* = 120) or right ventricular high septum (RVHS) (*n* = 120). At 2 years, LVEF decreased in both the RV apex (57 ± 9 to 55 ± 9%, *P* = 0.047) and the RVHS groups (56 ± 10 to 54 ± 10%, *P* = 0.0003) [38•]. However, there was no significant difference in intra-patient change in LVEF between confirmed RVA and RVHS lead position (*P* = 0.43) [38•]. Similarly, there were no significant differences in heart failure hospitalization, mortality, burden of atrial fibrillation, or plasma brain natriuretic peptide levels between the two groups [38•]. A significantly greater time was required to place the lead in the RVHS position (70 ± 25 vs. 56 ± 24 min, *P* < 0.0001) with longer fluoroscopy times (11 ± 7 vs. 5 ± 4 min, *P* < 0.0001). The authors concluded that in patients with high-grade AV block and preserved LV function requiring a high percentage of ventricular pacing, RVHS pacing does not provide a protective effect on LV function over RVA pacing in the first 2 years [38•].

His bundle pacing (HBP) is an alternative way to perform bradycardia pacing. The His-Purkinje conduction system allows the impulse generated by the sinoatrial node to rapidly propagate into both right and left ventricles which facilitates synchronized ventricular contraction. Early studies demonstrated distal HBP was able to normalize bundle branch block and QRS morphology [39]. The first successful series of permanent direct HBP was performed in 18 patients with atrial fibrillation (AF) and dilated cardiomyopathy in 2000 where the investigators found improvements in LV dimensions and cardiac function [40]. HBP may provide physiological activation thereby avoiding ventricular dyssynchrony and preserving LV systolic function in patients with a narrow QRS duration, and several studies have suggested a potential beneficial effect over RV pacing [41–45]. HBP may therefore be a way to avoid the potential deleterious effects of RV pacing; however, further randomized studies including The His Optimised Pacing Evaluated for Heart Failure (HOPE-HF) trial will be important in determining this.

There have been several studies examining CRT-based approaches to avoid the detrimental effects of apical RV pacing in patients with AV block and normal, mild, or moderate LVEF. PACE, PREVENT HF, and BLOCK HF have all directly compared CRT with RV pacing in patients with an indication for bradycardia pacing who were likely to require a high percentage of RV pacing (Table 1). These studies recruited patients in both sinus rhythm and AF. CRT has been shown to have advantages over RV pacing in four randomized clinical trials [14••, 46, 47, 53, 54]. There have also been smaller trials that have demonstrated an advantage of CRT pacing over RV pacing [33, 50, 51]. However, both BioPace and PREVENT HF have not been able to demonstrate a statistically significant benefit of CRT pacing over RV pacing in similar cohorts [48, 52••, 55]. All other trials included in Table 1 have shown CRT pacing to favor over RV pacing, irrespective of New York Heart Association (NYHA) class, baseline LV systolic function, degree of reverse remodeling, or QRS duration [26].

**Table 1:** Randomized clinical trials comparing right ventricular pacing and cardiac resynchronization therapy. CRT vs. RV pacing trials in patients requiring bradycardia pacing

Study	*n*	Inclusion criteria	Treatment	Follow-up	Endpoint	Results
PAVE [46]	184	Persistent AF and AV node ablation	CRT group (*n* = 81) RV group (*n* = 81)	6 months	LVEF 6MWT distance	RV group reduction 6MWT distance (*P* = 0.04) and LVEF (*P* = 0.03) vs CRT
Ablate and pace in AF [47]	186	Persistent/permanent AF	CRT (*n* = 97) RV (*n* = 89)	Median 20 months	Composite primary endpoint: death from HF, hospitalization for HF or worsening HF	Composite primary endpoint CRT 11% vs. RV group 26% (*P* = 0.005). CRT group less worsening HF (*P* = 0.0001) and less HF hospitalizations
DAVID [5]	506	Dual-chamber ICD indication	ICD VVI 40 bpm (back up pacing) (*n* = 256) ICD DDDR 70 bpm (*n* = 250)	Median 8.4 months	Composite primary endpoint: death from HF or first hospitalization for HF	1-year survival free of composite endpoint 83.9% patients with VVI-40 vs 73.3% for DDDR-70 (relative hazard, 1.61; 95% CI, 1.06–2.44)
MOST [23]	2010	PPM for sinus node dysfunction	Single-chamber VVIR pacing (*n* = 632) vs. DDDR pacing (707) for SND	Median 33.1 months	HF hospitalization and AF	RV pacing DDDR mode > 40% time led to 2.6-fold increased risk HF hospitalization vs. lower % pacing (normal baseline QRS duration, despite preservation of AV synchrony, in SND patients)
PREVENT HF [48]	108	Indication for pacing with LVEF > 50% and expected RV pacing of ≥ 80%	CRT (*n* = 50) RV apical (*n* = 58)	12 months	LVEDV	No significant difference between CRT and RV pacing in LVEDV. No change in LVEF, LVESV, or HF events
PACE [49]	177	LVEF ≥ 45% Standard bradycardia indications for pacing	CRT (*n* = 89) RV apical (*n* = 88)	Up to 2 years	LVESV LVEF	LVESV and LVEF deteriorated in RV apical group vs. no change CRT group, significant difference of 9.9% points between groups at 2-year follow-up (*p* < 0.001)
HOBIPACE [50]	30	Permanent RV pacing indication LVEDD ≥ 60 mm LVEF ≤ 40%	Run-in phase then randomized to 3 months RV pacing then 3 months CRT or vice versa	3 months with crossover to complimentary pacing mode	LVESV LVEF Peak oxygen consumption	Greater improvement in QoL, LVEF, maximal and submaximal exercise capacity CRT group vs. RV pacing group
COMBAT [51]	60	Standard RV pacing indication for AV block LVEF ≤ 40%, NYHA II–IV	Group A: RV pacing, then CRT, then RV pacing Group B: CRT, then RV pacing, then CRT	Minimum 3 months each mode	NYHA class and QoL score	In patients with systolic HF and AV block requiring permanent ventricular pacing, CRT was superior to RV pacing
BLOCK HF [14••]	691	AV block first to third HF NYHA I–III LVEF ≤ 50%	CRT (*n* = 349) RV pacing (*n* = 342)	Mean 37 months	Composite primary endpoint: time to death any cause, urgent care visit for HF requiring IV Rx, or ≥ 15% increase LVESV index	Primary outcome 190/342 pts. (55.6%) RV pacing group, vs. 160/349 pts. (45.8%) in CRT group. CRT group significantly lower incidence primary outcome vs. RV pacing group (HR, 0.74; 95% credible interval, 0.60–0.90)
BioPace preliminary results [52••]	1810	Indication for ventricular PPM according to guidelines or anticipated high frequency of V pacing	CRT (*n* = 902) RV pacing (*n* = 908)	Mean 5.6 years	Composite primary endpoint: first hospitalization due to heart failure or time to death	No statistically significant difference between CRT and RV pacing for composite primary endpoint (preliminary results)
Protect PACE [38•]	240	High-grade AV block requiring > 90% RV pacing with preserved LVEF > 50%	RV apical pacing (*n* = 120) RVHS pacing (*n* = 120)	2 years	Intra-patient change in LVEF	At 2 years, LVEF decreased in both RV apical (57 ± 9 to 55 ± 9%, *P* = 0.047) and septal groups (56 ± 10 to 54 ± 10%, *P* = 0.0003). No significant difference in intra-patient change LVEF between confirmed apical and septal lead position (*P* = 0.43)

*HF* heart failure, *LVEDV* left ventricular end-diastolic volume, *CRT* cardiac resynchronization therapy, *RV* right ventricular, *AF* atrial fibrillation, *CI* contraindication, *SND* sinus node dysfunction, *QoL* quality of life, *AV* atrioventricular, *LVESV* left ventricular end-systolic volume, *HR* hazard ratio

The Pacing to Avoid Cardiac Enlargement (PACE) trial was a prospective, double-blinded, randomized, multicenter study where patients with bradycardia and preserved LVEF were randomized to receive CRT (*n* = 89) or RV apical pacing (*n* = 88) [49]. Co-primary endpoints were LVEF and left ventricular end-systolic volume (LVESV) measured by 2D TTE. Patients were followed-up with a mean duration of 4.8 ± 1.5 years (2.5–7.8 years), and analyses of the primary endpoint were performed in 146 patients (CRT group *n* = 72, RV apical pacing group *n* = 74). The LVESV and LVEF remained unchanged in the CRT group whereas in the RV apical pacing group, not only did the LVEF decrease, the LVESV also increased progressively at follow-up [49]. The differences in LVEF between the RV apical pacing and CRT pacing groups were − 6.3% at 1 year, − 9.2% at 2 years, and − 10.7% at long-term follow-up (all *P* < 0.001). The corresponding differences in LVESV were + 7.4 milliliters (mL) at 1 year, + 9.9 mL at 2 years, and + 13.1 mL at long-term follow-up (all *P* < 0.001) [49]. In addition, the detrimental effects of RV apical pacing consistently occurred in all pre-defined subgroups (age groups, gender, QRS duration, pre-existing LV diastolic dysfunction, as well as pre-existing diabetes, hypertension, and coronary artery disease). Patients in the PACE trial with RV apical pacing had a significantly higher prevalence of heart failure hospitalization than the CRT group (23.9 vs. 14.6%, log-rank *χ*^2^ = 7.55, *P* = 0.006) [49]. The authors concluded that CRT was superior to RV apical pacing in the prevention of LV adverse remodeling and reduction of LVEF at 1 and 2 years follow-up. The Homburg Biventricular Pacing Evaluation (HOBIPACE) and the Conventional Versus CRT Pacing in Heart Failure and Bradyarrhythmia Therapy (COMBAT) studies were both small randomized studies that found CRT pacing superior to conventional RV apical pacing in terms of improvement in quality of life, exercise capacity, and LVEF as well as reduction in LV volumes [9, 50, 51]. HOBIPACE was a prospective, randomized crossover study where 30 patients, who had AV block, LVSD defined by a left ventricular end-diastolic diameter (LVEDD) ≥ 60 mm, and an LVEF ≤ 40% with NYHA II–IV, were randomized to 3 months of RV pacing then 3 months of CRT pacing or vice versa.

The COMBAT trial was a prospective, multicenter, randomized, double-blind crossover study that enrolled 60 patients with pacing indications for AV block with an LVEF < 40% and NYHA class II–IV for a mean follow-up period of 17.5 ± 10.7 months. All patients underwent CRT device implantation and were randomized to two groups and received the following for 3 months: Group A received RV pacing-CRT pacing-RV pacing and group B received CRT pacing-RV pacing-CRT pacing. There were significant improvements in LVEF, LVESV, NYHA class, and quality of life questionnaire scores in the CRT group compared to the RV pacing group. Death was more frequent with RV pacing; however, 6-min walk test (6MWT) distance and VO_2max_ were not significantly different between the two groups [51].

## BLOCK-HF and BioPace Studies

**Fig. 1:**
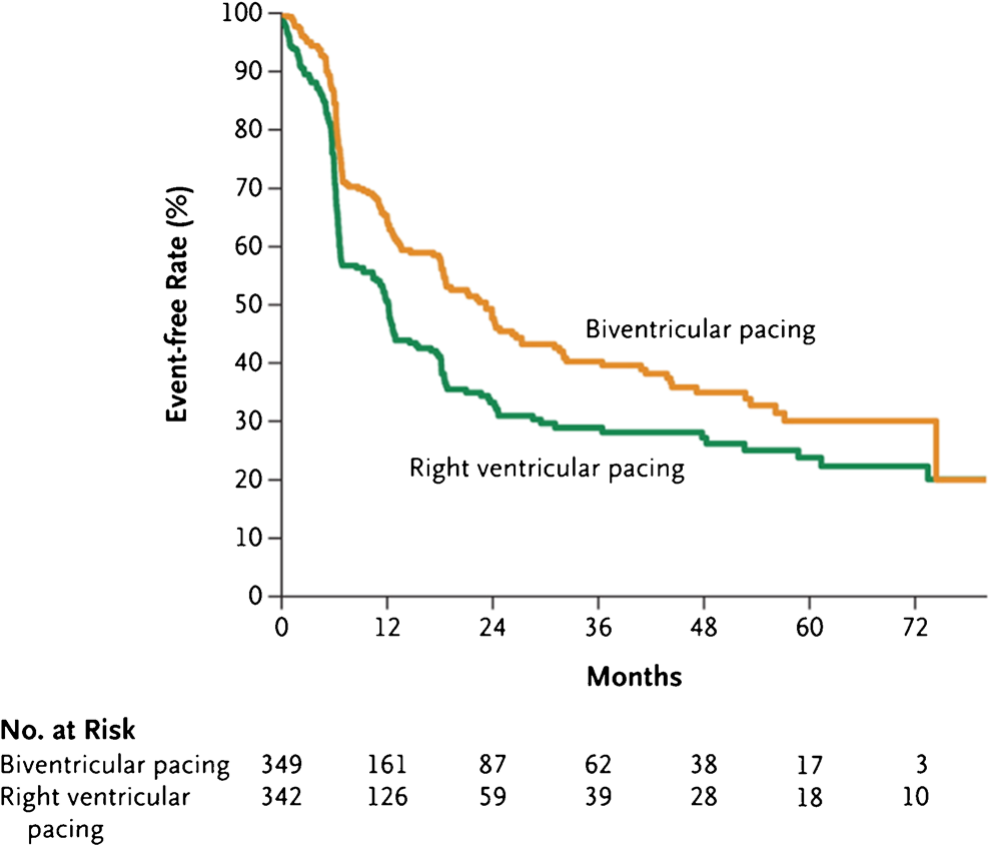
Freedom from composite primary endpoint (time to death from any cause, ≥ 15% increase in LVESV index, or an urgent care visit for heart failure that required intravenous therapy) in the BLOCK HF trial, copyright ^©^ 2013 Massachusetts Medical Society, reprinted with permission

To date, the most significant study to assess the benefits of CRT over RV pacing is the Biventricular versus Right Ventricular Pacing in Heart Failure Patients with Atrioventricular Block (BLOCK HF) trial. This was a large, multicenter, double-blind randomized study that assessed whether CRT reduced adverse LV remodeling, morbidity, and mortality in patients with AV block with a standard class I or IIa indication for ventricular pacing, NYHA class I–III heart failure, and LVEF ≤ 50% [14••]. Patients received a CRT pacemaker (CRT-P) unless they had an indication for defibrillation therapy in which case they received a CRT implantable cardioverter-defibrillator (CRT-D) and were randomized to receive either CRT pacing or standard RV pacing. Patients with standard indications for CRT, based on the guidelines during the recruitment phase, were excluded from recruitment as were patients with recent or acute myocardial infarction, unstable angina, percutaneous or surgical coronary revascularization within 30 days, or severe valvular heart disease with an indication for repair or replacement [14••, 15]. The primary outcome was time to death from any cause, ≥ 15% increase in LVESV index, or an urgent care visit for heart failure that required intravenous therapy. Nine hundred eighteen patients were enrolled, but only 691 patients underwent randomization in a 1:1 ratio. Patients were followed-up every 3 months with a mean follow-up duration of 37 months*.* The primary outcome occurred in 190 of 342 patients (55.6%) in the RV pacing group and 160 of 349 (45.8%) in the CRT group (hazard ratio, 0.74; 95% credible interval, 0.60 to 0.90). with a posterior probability of a hazard ratio < 1 was 0.9978, exceeding the threshold of 0.9775 for a significant different between the two groups (Fig. 1) [14••]. Similar findings were noted in patients receiving a CRT-P or CRT-D. Removing the echocardiographic volumetric indices from the analysis, death from any cause or an urgent care visit for heart failure still showed a significant difference in favor of CRT pacing compared to RV pacing with a hazard ratio of 0.73 (95% credible interval, 0.57 to 0.92) [14••, 15]. Of note, 6.4% of patients had a complication documented secondary to LV lead implantation. A subsequent substudy of BLOCK HF demonstrated reverse remodeling within the CRT group using 2D TTE, where CRT pacing significantly reduced intraventricular mechanical delay and LV volume indices along with improvement in LVEF compared to RV pacing, all indicating LV reverse remodeling. The risk of morbidity and mortality was estimated to increase by up to 1% for every 1 mL/m^2^ increase in LVESV index, suggesting LVESV index may be predictive of morbidity and mortality [56]. The main limitation of BLOCK HF was a high crossover rate from the RV pacing group to CRT group as well a reasonably large amount of missing 2D TTE data.

The preliminary results of the Biventricular Pacing for Atrioventricular Block to Prevent Cardiac Desynchronisation (BioPace) trial were announced in 2014 [52••, 55]. BioPace was a multicenter, randomized, single-blind study conducted in Europe and aimed to investigate the hypothesis that CRT pacing is superior to RV pacing in patients with AV block requiring permanent ventricular pacing. The combined primary endpoint was first hospitalization secondary to heart failure or time to death. Main inclusion criteria were patients with an indication for implantation of a ventricular pacemaker according to European Society of Cardiology (ESC) guidelines and an anticipated need for frequent ventricular pacing with any LVEF as measured by TTE. Patients with first, second, and third AV block were enrolled. For first-degree AV block, the defining PR interval was ≥ 220 milliseconds (ms) with an indication for pacing. Patients with permanent AF were also included providing their spontaneous ventricular rate was ≤ 60 beats/min at rest. One thousand eight hundred ten patients were recruited, and 902 patients were assigned to the CRT group and 908 to the RV pacing group. The patient demographics were largely similar to BLOCK HF except the average LVEF in BioPace was 55% compared to approximately 40% in Block HF. The preliminary results from BioPace showed no statistically significant difference between CRT pacing and RV pacing for first hospitalization secondary to heart failure or time to death. However, there was a non-significant trend in favor of CRT pacing vs. RV pacing. Additional analyses might identify subgroups of patients where CRT pacing shows a clear and statistically significant benefit. Interestingly, LVEF did not seem to have any influence on the combined primary outcome as the results were similar for LVEF ≤ 50 vs. > 50%. It is not immediately obvious why the BioPace study results differed to BLOCK HF; however, different patient demographics are likely to have played a role. Furthermore, patients in the BLOCK HF trial had a greater number of patients with LBBB (total 32.6%, CRT pacing group 35.2%, RV pacing group 29.8%) compared to BioPace (total 17.2%, CRT group 16.6%, RV group 18.3%) and lower LVEF, possibly indicating a cohort with more severe heart failure. Furthermore, AF is a recognized marker for underlying morbidity, and again more patients in the BLOCK HF trial had AF (total 52.8%, CRT group 51.6%, RV group 54.1%) vs. BioPace (total 24.9%, CRT group 24.9%, RV group 24.8%) indicating the higher morbidity in the BLOCK HF cohort. The long-awaited final published results from the BioPace investigators may help to better understand the results and differences to the BLOCK HF trial.

## The Role of CRT in Patients With Atrial Fibrillation Undergoing AV Node Ablation

There have been several studies that have demonstrated better outcomes with CRT followed by AV node ablation than RV pacing in patients with symptomatic atrial fibrillation with rapid ventricular response [46, 47, 57–59]. In 2012, a meta-analysis of the aforementioned studies as well as two other similar studies found CRT pacing was associated with a significant reduction in hospitalizations for heart failure (RR = 0.38, 95% CI = 0.17–0.85; *P* = 0.02). Moreover, they established a non-significant reduction in mortality compared to RV pacing (RR = 0.75, 95% CI = 0.43–1.30; *P* = 0.30) [59]. Conversely, there was no significant difference in Minnesota Living With Heart Failure Questionnaire Score or 6MWT distance between CRT and RV pacing groups. In 2010, Orlov et al. randomized 153 patients in a single-blinded trial and revealed a significant increase improvement in LVEF in the CRT pacing group; however, in the RV pacing group, there was a non-significant reduction in LVEF [58]. Similarly, Brignole et al. conducted a prospective, multicenter study (The Ablate and Pace in AF Trial) and randomized 186 patients who had undergone CRT device implantation and AV node ablation to receive either CRT (*n* = 97) with V-V interval optimization or RV apical pacing. Baseline demographics were similar to the PAVE study, and follow-up was a median of 20 months (interquartile range 11–24). The primary composite endpoint of death from heart failure, hospitalization due to heart failure, or worsening heart failure occurred in 11% patients in the CRT group and 26% patients in the RV group [CRT vs. RV group: subhazard ratio (SHR) 0.37 (95% CI 0.18–0.73), *P* = 0.005] [47]. Fewer patients had worsening heart failure in the CRT group compared to the RV group [SHR 0.27 (95% CI 0.12–0.58), *P* = 0.001] and fewer hospitalizations for heart failure [SHR 0.20 (95% CI 0.06–0.72), *P* = 0.013] [47]. There was, however, no significant difference in total mortality, although the authors concluded that CRT was superior to RV apical pacing in reducing the clinical manifestations of heart failure in patients requiring an AV node ablation for symptomatic AF [47]. The Left Ventricular-Based Cardiac Stimulation Post AV Nodal Ablation Evaluation (The PAVE study) was a prospective randomized controlled study that compared CRT pacing with RV pacing in 184 patients with NYHA functional class I to III heart failure (baseline LVEF 45% ± 15% in the CRT group vs. 47% ± 16% in the RV group) undergoing an AV node ablation for atrial fibrillation (AF) refractory to pharmacotherapy [46]. Patients undergoing ICD implantation were excluded. The PAVE study showed that patients randomized to CRT (*n* = 103) had significant improvements in LVEF and 6MWT but not in quality of life parameters compared to the RV paced group. At 6 months post ablation, patients treated with CRT had a significant degree of improvement in 6MWT, 31% above baseline (82.9 ± 94.7 m), compared to patients receiving RV pacing, 24% above baseline (61.2 ± 90.0 m) (*P* = 0.04) [46]. At 6 months post ablation, the LVEF in the CRT group (46 ± 13%) was significantly greater in comparison to the RV pacing group (41 ± 13%, *p* = 0.03) [46]. The LVEF remained stable for patients in the CRT group whereas in the RV pacing group, the LVEF deteriorated by 3.1% at 6 weeks (*P* = 0.04) and 3.7% at 6 months (*P* = 0.03) [46]. The authors concluded that CRT provided a significant improvement in 6MWT distance and LVEF compared to RV pacing in patients undergoing AV node ablation for AF. Furthermore, patients with LV systolic impairment or symptomatic heart failure derived the greatest benefit from CRT pacing [46].

BLOCK HF was a landmark US-based trial that revealed encouraging evidence that improved outcomes may be achieved with CRT pacing compared to RV apical pacing in patients with LVSD and AV block when a high percentage of RV pacing is anticipated [9, 15]. As a result, in 2014, the United States Food and Drug Administration approved the use of CRT in patients with AV block associated with a high percentage of ventricular pacing, mild to moderate heart failure, and LVEF ≤ 50% [60]. In 2016, the ESC guidelines for the diagnosis and treatment of acute and chronic heart failure were updated recommending CRT over RV pacing for patients with high-degree AV block, heart failure with reduced ejection fraction (HFrEF), and NYHA I–IV functional class in order to reduce morbidity (1A evidence, Table 2) [61••]. Patients with AF were included in this guidance.

**Table 2:** Summary of 2016 ESC Guidelines for the diagnosis and treatment of acute and chronic heart failure relating to CRT and RV pacing in patients with high-degree AV Block. *Adapted from 2016 ESC Guidelines for the Diagnosis and Treatment of Acute and Chronic Heart Failure* [61••]

ESC recommendation	Class	Level
CRT is recommended over RV pacing for patients in sinus rhythm or AF, with HFrEF of any NYHA functional class, who have an indication for ventricular pacing and high-degree AV block, in order to reduce morbidity.	I	A
CRT is recommended over RV pacing in patients with HFrEF who require pacing with a high-degree of AV block.	I	A
Pacing modes that avoid inducing or worsening ventricular dyssynchrony should be considered for patients with HFrEF who require ventricular pacing without high-degree AV block.	IIa	C

*ESC* European Society of Cardiology, *CRT* cardiac resynchronization therapy, *HFrEF* heart failure with reduced ejection fraction, *NYHA* New York Heart Association, *AV* atrioventricular, *RV* right ventricular

## Conclusions

The role of CRT pacing in patients with AV block and impaired LV systolic function remains an important consideration. The BLOCK HF trial demonstrated better outcomes with CRT pacing over RV pacing in patients with LVSD and AV block in patients expected to have a high RV pacing burden. However, BLOCK HF failed to demonstrate any mortality benefit. The preliminary results of the European-based BioPace trial have not confirmed the same statistically significant benefit, although we are still awaiting the full results to be published. In the interim, CRT pacing seems to have a beneficial effect on LV reverse remodeling, systolic function, and clinical outcomes in patients with NYHA functional class I–III heart failure, moderate to severe LVSD, and AV block compared to RV pacing. However, it is less clear whether there is a similar benefit from CRT in patients with a high percentage of RV pacing who have normal or mild LVSD in the treatment of AV block.

## Abbreviations

2D: 2-dimensional
3D: 3-dimensional
6MWT: 6-min walk test
AV: Atrioventricular
CRT: Cardiac resynchronization therapy
CRT-D: Cardiac resynchronization therapy defibrillator
CRT-P: Cardiac resynchronization therapy pacemaker
ESC: European Society of Cardiology
HBP: His bundle pacing
HF: Heart failure
ICD: Implantable cardioverter-defibrillator
LBBB: Left bundle branch block
LV: Left ventricular
LVEDD: Left ventricular end-diastolic dimension
LVEF: Left ventricular ejection fraction
LVESV: Left ventricular end-systolic volume
LVSD: Left ventricular systolic dysfunction
ms: Milliseconds
NYHA: New York Heart Association
RV: Right ventricular
RVHS: Right ventricular high septum
TTE: Transthoracic echocardiogram
vs.: Versus
